# Phosphorus Paradox Scarcityand Overabundance of a Key Nutrient

**DOI:** 10.1289/ehp.119-a208

**Published:** 2011-05

**Authors:** Tim Lougheed

**Affiliations:** **Tim Lougheed** has worked as a freelance writer in Ottawa, Canada, since 1991. A past president of the Canadian Science Writers’ Association, he covers a broad range of topics in science, technology, medicine, and education

In between shepherding the United States through the Great Depression and bracing the country for war in Europe, then-President Franklin Roosevelt took a moment in 1938 to tell Congress about phosphorus.^1^ More specifically, he spoke about phosphates, the most commonly commercially exploited form of phosphorus.

Roosevelt’s was not a scientific presentation but a cautious alert about the critical role this element plays in agricultural production. Private interests were exporting increasing amounts of the country’s phosphates to markets abroad, he warned. Given the prospect that this vital constituent of fertilizer could come into short supply domestically, Roosevelt recommended framing a formal policy to deal with a strategic issue.

“The disposition of our phosphate deposits should be regarded as a national concern,” he said. “The situation appears to offer an opportunity for this nation to exercise foresight in the use of a great national resource heretofore almost unknown in our plans for the development of the nation.”^1^

In spite of Roosevelt’s call, no phosphate policy was forthcoming. More than seven decades on, the phrase “heretofore almost unknown” still echoes in many discussions about phosphorus. Meanwhile, the significance of phosphates is even more profound today than it was in the 1930s. Phosphate rock has emerged as a globally traded commodity linked to a diverse set of politically charged debates, ranging from environmental degradation and threats to human health to food security and agricultural sovereignty.

## “Life’s Bottleneck”

Phosphorus, among the most common elements found in the Earth’s crust,^2^ was dubbed “life’s bottleneck” by science writer Isaac Asimov. “[L]ife can multiply until all the phosphorus is gone, and then there is an inexorable halt which nothing can prevent,” he wrote. “We may be able to substitute nuclear power for coal, and plastics for wood, and yeast for meat, and friendliness for isolation—but for phosphorus there is neither substitute nor replacement.”^3^

Alfalfa, Asimov noted, could thrive in soil made up of 0.1% phosphorus, while the plant’s structure consisted of 0.7% phosphorus. This stoichiometric need for phosphorus makes the element not only a governing factor in plant growth but an irreplaceable one. No known input—natural or synthetic— can stand in for phosphorus.

Even though phosphorus was chemically identified only a few centuries ago, it has been employed throughout agricultural history in the crop residues and manure spread on fields. This traditional practice continues, but an increase in phosphorus mining throughout the twentieth century contributed to steadily rising agricultural yields. Fertilizers manufactured with high proportions of phosphorus, nitrogen, and potassium boosted plant growth to unprecedented levels, especially in tropical soils that are poor in these constituents.^4^

Well before the celebrated Green Revolution that began in the 1940s before really taking off in the 1960s, manufactured fertilizer was gearing up farmers to feed more people than the world had ever known. Doomsayers arguing that a billion of us were too many at the beginning of the twentieth century would certainly have been awestruck to see more than 6 billion humans alive and mostly well by the turn of the millennium.

And agricultural output has not just kept pace with steady population growth, but gained on it. In 2010, as the global population closed in on 7 billion, the Food and Agriculture Organization of the United Nations reported an absolute decline in the number of undernourished people in the world over the preceding year—although at 925 million souls that figure is still staggering.^5^

Phosphorus is seldom credited for this decline, but such progress would have been unthinkable without its dramatically expanded use in the form of phosphate-based fertilizer.^4^ As Roosevelt’s address to Congress indicated, the importance of this resource was well established by the 1930s. Global production of phosphate rock is now nearly 13 times what it was then,^6^ although estimates of the amount left in the ground vary, according to Dana Cordell, a research principal at the Institute for Sustainable Futures at the University of Technology, Sydney, and co-founder of the Global Phosphorus Research Initiative.

Cordell carried out one of the more determined attempts^7^ to pin down those numbers. To hear her describe it, researchers in her field regard phosphorus as no obscure biochemical bottleneck but an agent that should occupy the center stage of many different scientific and policy discussions.

“Phosphorus plays many roles in society today—both desired and undesired,” she wrote in 2010. “At any moment in time, phosphorus fulfils numerous different functions—on vastly different temporal and geographical scales: transporting split-second signals to the brain in the chemical ATP, or immobile as a Ca_3_(PO_4_)_2_ molecule in apatite-rich phosphate rock that took tens of millions of years to form, awaiting extraction, or gradually being drawn up from soil solution by plant roots via chemical diffusion, or discharging from our bodies in a momentary drop of urine before being diluted by a flood of flush water to join other household and industry wastewater at a distant treatment plant, polluting water bodies as cyanobacteria, or simply cycling naturally between land, biota and water without being noticed by most of society.”^8^

## Too Much of a Good Thing

Despite its virtues as an elemental staff of life, phosphorus has also earned a reputation as a pollutant. In rural areas it regularly flows into receiving water as runoff from agricultural fields,^9^ and in urban areas from sewage sources as a major constituent of human excreta flushed down toilets. In either instance, phosphorus can excessively boost local nutrient levels, promoting algal blooms in the lakes and rivers where it concentrates—a process called eutrophication.^10^

This excessive algal growth can eventually lower oxygen levels in the water to the point where some fish species can no longer survive. Such was the condition that afflicted Lake Erie in the 1970s, attracting the attention of University of Alberta biologist David Schindler. Using a small test lake in northern Ontario that was divided in two with a submerged curtain, he demonstrated that the phosphate detergents emerging from municipal wastewater streams were a major driver of Lake Erie’s problem.^11^ Detergent manufacturers were subsequently persuaded to severely limit the amount of phosphates in their products, which substantially reduced the amount of eutrophication in the lake.^12^

Since then, Schindler has continued to study the ongoing challenge that nutrient overload poses to aquatic environments and human health. “Clearly, biological waste disposal activities such as manure applications to cropland can simultaneously increase the loading of phosphorus, nitrogen, and potentially hazardous coliform bacteria to surface waters,” he and coauthor Val Smith wrote in 2009.^13^ “However, enhanced nutrient loading alone might also influence the abundance, composition, virulence and survival of pathogens that are already resident in aquatic ecosystems.”

Nor was Lake Erie alone in its biochemical challenge, according to the International Lake Environment Committee (ILEC), a Japanese nongovernmental organization that has been surveying the health of 217 large freshwater bodies since 1986. In five publications between 1988 and 1994, ILEC reported on lakes around the world that faced much the same problem in the second half of the twentieth century. While local action eventually lowered input levels at 66 of those lakes, ongoing data^14^ indicate that all of them retain far higher nutrient levels than they had several decades ago.

The impact of this change is far more than aesthetic. A 2009 review in *Environmental Science & Technology* put the annual price tag of eutrophication in the United States at a conservative $2.2 billion.^15^ While compromised waterfront property values and lost recreational opportunities made up much of this total, the researchers assigned $813 million to a demand for bottled water created by the unacceptable taste and odor of drinking water that would otherwise be drawn from eutrophied sources. “This estimate is based purely on bottled drinking water costs and does not take into account additional costs related to alternative drinking water treatments such as wells or hauling drinkable water from another area,” state authors Walter K. Dodd and colleagues, who drew on a survey of 241 water facilities.

As for other health-related costs, the authors state, “For humans, algal blooms cause sicknesses and rarely result in death. We did not include human health costs because they appear to be minor compared to other factors we investigated. Still, people might be more likely to spend considerable amounts to avoid toxic blooms.”^15^

Even in its beneficial application as fertilizer, phosphorus may impose a human health cost. For instance, depending on where phosphate rock deposits occur, they may coexist with varying amounts of heavy metals. A 2010 report commissioned by the EU Directorate-General for the Environment and undertaken jointly by Wageningen University and Stockholm Environment Institute expresses immediate concern over cadmium,^16^ a well-established renal, bone, and pulmonary toxicant that occurs naturally in phosphate rock deposits.^17^ Phosphate fertilizers are considered the main source of cadmium in agricultural soils.^18^

The particular amount of cadmium that is found in any given phosphate deposit can vary widely from one part of the world to another. For example, in the igneous geology of South Africa there may be just 0.04–4.0 mg cadmium for each kg phosphorus, while in the sedimentary layers of Senegal that proportion jumps to 71–148 mg/kg.^16^

The Directorate-General report acknowledges an International Fertilizer Industry Association conclusion that the slow accumulation of cadmium in agricultural soils around the world has not produced concentrations high enough to warrant action.^19^ That conclusion was subsequently reinforced by a two-year field study of the transfer rates of cadmium in fertilizer to lettuce growing in the same soil, published in *Water, Air and Soil Pollution* in two parts in 2004.^20^,^21^ This research revealed that the use of triple superphosphate actually decreased the efficiency of cadmium transfer to the soil over the longer term.

However, a similar study of transfer rates in potatoes, published in the same journal a year earlier, cautioned against the aggressive use of phosphate-intense fertilizers. “Zones that were found to have high heavy metal levels should be avoided to cultivate potatoes because of the fact that potatoes tend to accumulate heavy metals notably higher than other types of plants,” wrote Emine Erman Kara and colleagues. “Soils that were found to be acidic should be treated with lime so that heavy metal uptake by plants via soil-plant pathway could be slowed. Moreover, it could also prevent groundwater resources in the region from heavy metal contamination especially in acidic zones.”^22^

The 2010 Directorate-General report also cites a 2000 report to the European Commission suggesting that cadmium levels in fertilizers could warrant regulations stipulating that phosphates for fertilizers used within the EU undergo treatment to remove virtually all traces of the metal.^23^ This process, which uses heat to eliminate cadmium, is feasible but expensive.^24^,^25^ This means producing a more health-friendly fertilizer may limit accessibility to essential fertilizers for farmers who lack the means to purchase them.^26^

## Not Enough of a Good Thing

That lack of accessibility to fertilizer is already a reality for farmers in countries without significant phosphate resources. There are two separate but interrelated issues here, Cordell says. First, poor farmers working with phosphorus-deficient soils cannot access fertilizer markets, particularly in sub-Saharan Africa. Second, only a few countries control the world’s remaining phosphate reserves, which makes any country that depends on imports vulnerable to volatility in price and availability.^7^

Such countries are numerous, as the world’s largest deposits of this material are thought to be relatively few. A 2010 estimate by the U.S. Geological Survey (USGS)^27^ identified fully 93% of the world’s phosphate reserves as belonging to just six countries—Morocco, China, Algeria, Syria, South Africa, and Jordan—with more than 83% of the global total found in Morocco alone.

A more recent estimate by the International Fertilizer Development Center (IFDC), based on a literature survey, dramatically raised that global total to more than three times the amount suggested by the USGS.^28^ Most of that increase was made up of reserves in Morocco, which was found to contain fully 85% of the global total. The author of the IFDC report, Steven van Kauwenbergh, acknowledged the substantial disparity between these two estimates as well as the lack of reliable, publicly available data, which prompted him to call for a more comprehensive approach to the subject by a much broader field of observers than members of the phosphate industry.

“A collaborative effort by phosphate rock producers, government agencies, international organizations and academia will be required to make a more definitive estimate of world phosphate resources,” he argued in the report.^28^ Van Kauwenbergh further insisted that despite the limitations of the data presented in the report, the current rate of fertilizer production could be maintained for several centuries.

That prediction does not satisfy everyone, however. In an April 2011 critique of the IFDC report, Cordell and colleagues challenged the assumptions both that 100% of the reserve is accessible and that consumption will not increase. Moreover, they wrote, “There is consensus that the world’s remaining phosphate reserves are declining in phosphorus concentration, increasing in impurities and becoming harder to physically access. Meanwhile, phosphate extraction increasingly generates more pollution and waste, requires more energy per nutrient value and costs more to mine and to process.”^29^

James Elser, who began his career studying aquatic life, has become intensively interested in how energy and specific chemicals move through the environment. Phosphorus now looms large in his integrative work on biological stoichiometry, leading Elser to focus on how our zealous use of the element has disrupted its natural flow through the environment.

Elser and a handful of colleagues at Arizona State University founded the Sustainable Phosphorus Initiative to raise awareness of the questions that persist about the long-term supply of this commodity. In February 2011, their efforts resulted in the Sustainable Phosphorus Summit,^30^ a three-day gathering of more than 100 scientists, engineers, farmers, and entrepreneurs.

“The summit was distinguished by its participatory, interdisciplinary, and creative approach that allowed participants from diverse backgrounds to share their different knowledge and perspectives on the global phosphorus challenge,” says Cordell, who spoke at the event. “It was very solution-focused and came up with strategies for how we might move together toward a more sustainable situation.”

Those strategies could include minimizing the amount of phosphorus used in agriculture, recovering any runoff before it enters the environment, and even recycling it for future use.^31^ And, adds Elser, the search for more coordinated approaches to the environmental management of phosphorus cannot come too soon. “We’re in charge of the phosphorus cycle now,” he points out. “The fluxes that we generate are larger than natural fluxes. This is no way to run a biogeochemical cycle.”

## Figures and Tables

**Figure:**
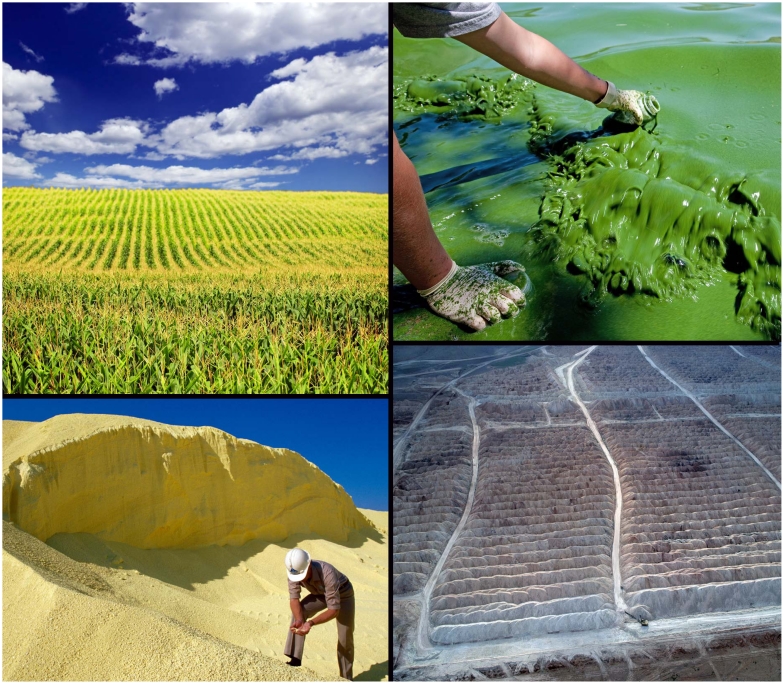
Faces of phosphorus (clockwise from top right): a California lake smothered in cyanobacteria; a phosphate mine near Casablanca, Morocco; a fertilizer plant in Australia; a cornfield approaching harvest. Clockwise from top right: © David McLain/Aurora Photos/Corbis © Yann Arthus-Bertrand/Corbis © Robert Garvey/Corbis © Elena Elisseeva/Shutterstock

**Figure:**
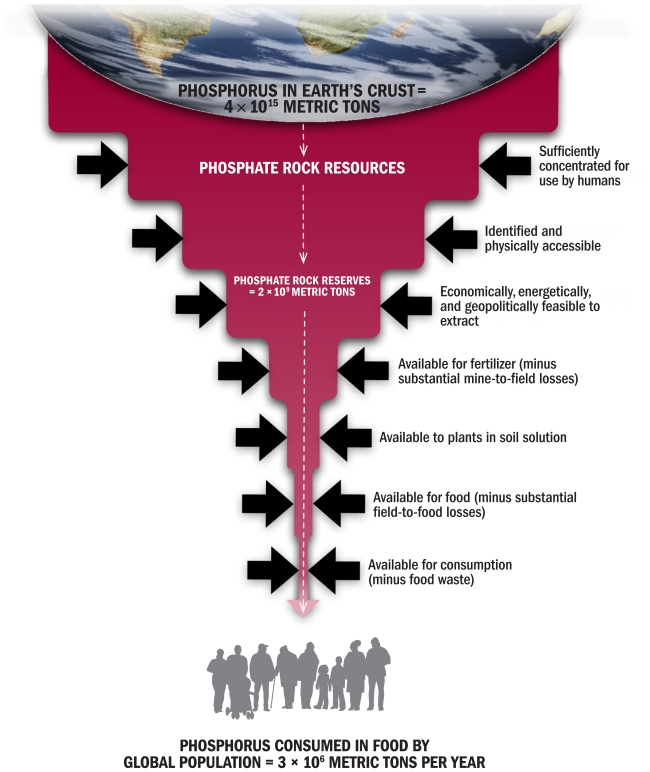
Although phosphorus is one of the most common elements on Earth, only a small percentage is available for human use. (Source: Schröder et al.^16^)

**Figure:**
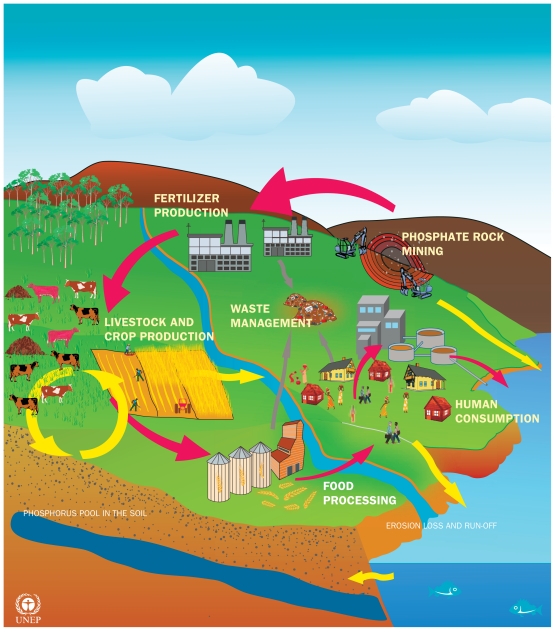
Red arrows show the primary direction of phosphorus flows. Yellow arrows show the recycling of phosphorus in the crop and soil system and movement toward water bodies. Gray arrows show phosphorus lost through food wastages in landfills. (Source: UNEP^32^)
